# Oral Health and Behavior Patterns of Women with Eating Disorders—A Clinical Pilot Study

**DOI:** 10.3390/life13122297

**Published:** 2023-12-03

**Authors:** Mojdeh Dehghan, Daranee Tantbirojn, Janet Harrison, Colette W. Stewart, Nancy Johnson, Elizabeth A. Tolley, Yanhui H. Zhang

**Affiliations:** 1 College of Dentistry, University of Tennessee Health Science Center, 875 Union Avenue, Memphis, TN 38163, USA; 2 Department of General Dentistry, College of Dentistry, University of Tennessee Health Science Center, 875 Union Avenue, Memphis, TN 38163, USA; 3 Department of Bioscience Research, College of Dentistry, University of Tennessee Health Science Center, 875 Union Avenue, Memphis, TN 38163, USA; 4 Transformation Center, 1088 Rogers Road, Cordova, TN 38018, USA; 5 Department of Preventive Medicine, College of Medicine, University of Tennessee Health Science Center, 66 N Pauline, Memphis, TN 38163, USA

**Keywords:** behavior, dental caries, eating disorders, saliva, tooth erosion

## Abstract

Background: Chronic stomach regurgitation associated with eating disorders (EDs) poses a high risk for tooth erosion. This study investigated oral health conditions, behavioral patterns, and tooth erosion in women with EDs. Methods: 16 ED and 13 healthy women were enrolled; 14 ED and 10 healthy control subjects completed the study. Subjects completed demographic, medical, oral, and behavioral health history questionnaires. Dental caries status was recorded as Decayed, Missing and Filled Teeth (DMFT)index and the severity of tooth erosion as Basic Erosive Wear Examination (BEWE) scores. Saliva was collected for flow rate, pH, and buffering capacity analysis. Results: The ED group had a lower stimulated saliva flow rate and higher DMFT index but no significant difference in BEWE scores compared to the controls (t-test, significance level 0.05). Five of the fourteen ED subjects exhibited extensive tooth erosion, which may have been exacerbated by their tooth-brushing behavior. Conclusions: Although some ED subjects showed extensive tooth erosion in this pilot study, the average BEWE score of the ED group was not significantly different from the controls. Extensive tooth erosion in ED may relate to the low stimulated salivary flow. A larger-scale clinical study is necessary to validate these results.

## 1. Introduction

Practicing dentists who have treated patients with advanced tooth erosion caused by eating disorders (ED) will agree that the destruction of tooth structure is often extensive and requires costly dental treatment. Dental erosion, a form of tooth wear caused by intrinsic or extrinsic acid dissolving tooth structures, can lead to tooth pain from hypersensitivity and loss of the form and function of the dentition, compromising aesthetics, and even causing pulpal damage that requires root canal treatment [1]. The mineralized tooth substance is dissolved by exposure to acids of non-bacterial origin [2,3]. Hydrochloric acid, as found in gastric juice, causes tooth erosion when the gastric refluxate enters the oral cavity. Erosive tooth wear is a growing oral health concern, partly due to changes in lifestyle [1]. Globally, the prevalence of erosive tooth wear in permanent teeth ranged from 20% to 45% [4]. The prevalence of erosive tooth wear in the United States was 79.8% among adults 20–65 years old, estimated from the 2003–2004 National Health and Nutrition Examination Survey [5].

Patients with EDs are more prone to tooth erosion due to self-purging of stomach contents manifested as vomiting [6,7,8,9]. Dental erosion due to repeated stomach acid exposure is the most frequent oral manifestation of ED, classified as bulimia nervosa [6]. ED patients with vomiting or binge eating behaviors were 5.5 times more likely to have erosive tooth wear than those without such behavior [8]. The number of years of binge eating contributed to the presence of erosive tooth wear; the longer the duration of the disease, the more common the tooth erosion [8,10]. More than half of the 81 subjects with eating disorders (seventy-nine females and two males) had erosive tooth wear extending beyond the enamel, and about one-third had very low unstimulated salivary flow rates [10]. An epidemiological study review reported a 2 percent lifetime prevalence of bulimia nervosa among women [11]. According to the National Eating Disorders Association, national surveys estimate that 28.8 million Americans in the United States will have an ED at some point [12]. Unfortunately, since EDs are mental health conditions and carry a negative social stigma, many patients are reluctant to disclose their condition to medical and dental professionals [13]. Consequently, a growing population with EDs is underrepresented, underdiagnosed, and undertreated.

Dental erosion is a multifactorial condition with biological, behavioral, and chemical risk factors that modulate its prevalence and progression [1]. In a healthy individual, when intrinsic or extrinsic acid dissolves (demineralizes) tooth enamel, saliva can remineralize the affected structure [14]. ED patients may show an accelerated rate of tooth erosion, with frequent exposure to intrinsic acid being the simplest explanation for the risk factor. A clinical trial showed that key characteristics of saliva after vomiting in ED patients, including the presence of certain enzymes, flow rate, pH, and buffering capacity, affected tooth erosion [15]. Risk factors can predict the rate of dental erosion [16]. Thus, knowledge about risk factors will better prepare dental professionals to recommend and provide a suitable preventive regimen for patients.

Collaborative efforts between mental health care professionals and the dental team can increase the likelihood of early detection and treatment of tooth erosion in patients with eating disorders [17]. Dentists and dental hygienists can be the only medical professionals who routinely examine patients with eating disorders, and are in a unique position to diagnose and refer these patients to appropriate medical and psychological experts [18]. The immense need for dental care of patients with ED is recognized, but information about this patient population is limited [19,20]. This study investigates oral health conditions, behavior, and saliva characteristics related to tooth erosion in a subpopulation of women suffering from EDs. The two hypotheses tested were (1) the severity of tooth erosion in adult females with ED is greater than in healthy women, and (2) the specific health history, oral health behavior, oral conditions, and saliva characteristics are predisposing risk factors for tooth erosion.

## 2. Materials and Methods

### 2.1. Study Design

**Figure 1:**
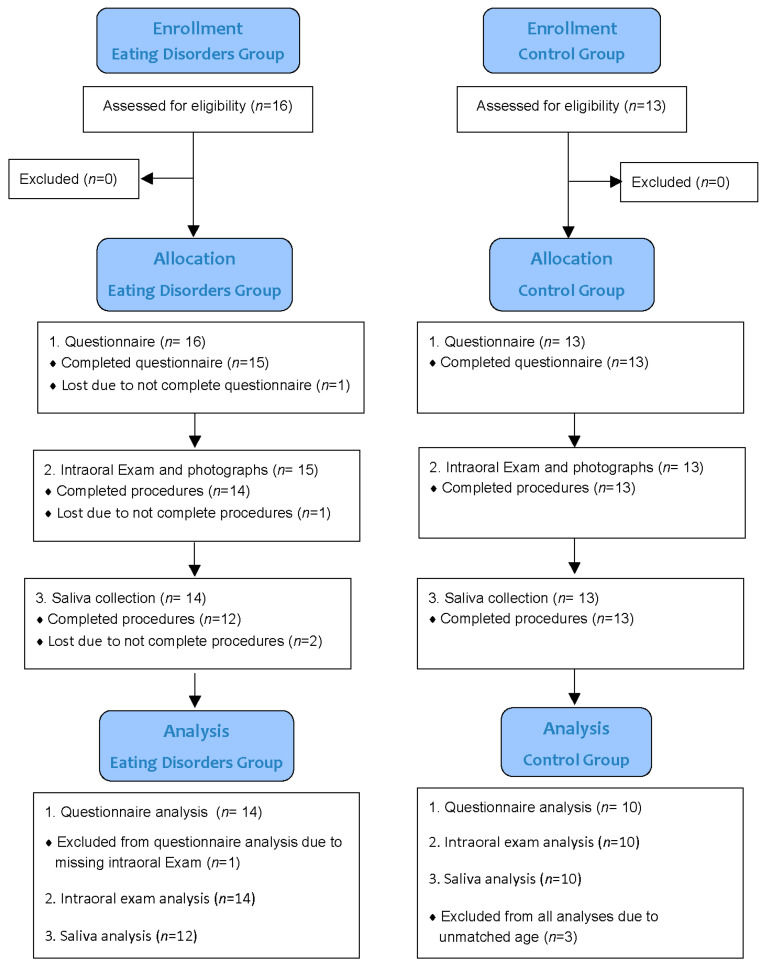
CONSORT Flow Diagram.

This pilot clinical study investigated oral care behavior and oral health characteristics of subjects with EDs through a survey (questionnaire), clinical examination of teeth, and saliva analysis. The STROBE (Strengthening the Reporting of Observational studies in Epidemiology) Statement for cross-sectional studies was followed. Subjects completed all study sessions within one visit or returned to finish on the second visit. Figure 1 is a flow diagram of the study design.

### 2.2. Subject Recruitment and Background Information

After the University of Tennessee Health Science Center (UTHSC) Institutional Review Board (Memphis, TN, USA) approval (15-03995-XP), 16 subjects with EDs were recruited from the Transformation Center (Cordova, TN, USA), an outpatient rehabilitation center for ED patients. Of the 16 ED subjects enrolled, 12 completed the study and 4 dropped out (2 have survey and oral examination data). For a control group, 13 healthy female subjects were recruited at the dental school clinical research center. Among them, 10 subjects completed the study, and 3 were disqualified because their ages were not within the age range of ED subjects. Subjects were at least 18 years old. Inclusion criteria for the ED group were being diagnosed with eating disorders and having at least 16 natural teeth with a minimum of four teeth in each quadrant. Subjects diagnosed with eating disorders at the Transformation Center went through a thorough psychosocial assessment that met the criteria DSM-5 for diagnosis of an eating disorder [21]. They were reviewed and approved by the Clinical Director (a mental health service provider and certified eating disorder specialist) and the Medical Director. Control subjects were qualified if they answered ‘no’ to all of the criteria for ED. Exclusion criteria were being pregnant or lactating; taking antibiotics, immune-suppressive, or other drugs; or using anti-gingivitis and/or antibacterial oral care products 2 weeks before the visit. The subjects signed the written informed consent form and were informed about confidentiality following the HIPAA Standards for Privacy of Individually Identifiable Health Information guidelines. Subjects refrained from eating, drinking, and practicing oral hygiene after 9 p.m. the night before the saliva collection visit.

### 2.3. Questionnaire: Eating Disorders and Oral Care Behaviors

Subjects completed a 3-page questionnaire to collect information about their health history and behaviors. The questionnaire covered (a) the general patient profile (age, height, weight, etc.); (b) eating and drinking behaviors; (c) smoking, alcohol, and drug use; (d) ED duration and purging behaviors; (e) oral hygiene practice and oral care; and (f) medications and specific medical conditions.

### 2.4. Intraoral Examination

Two clinical examiners were trained and calibrated to perform a visual oral examination. They recorded visible caries, fillings, crowns, and missing teeth according to the World Health Organization (WHO, Geneva, Switzerland) criteria for the Decayed, Missing and Filled Teeth (DMFT) index [22]. The presence of tooth erosion was recorded following Lussi’s scoring system for Basic Erosive Wear Examination (BEWE) by grading the facial, lingual, and occlusal/incisal surfaces for severity on a four-point scale, where 0 = no sign of erosion; 1 = initial enamel erosion; 2 = moderate enamel erosion; and 3 = erosion involving dentin [23]. Intraoral photographs were obtained.

### 2.5. Saliva Collection and Analysis

Resting and stimulated saliva were collected between 8 a.m. and noon. Saliva flow rate, pH, and buffering capacity were determined immediately after collection. For the resting saliva, subjects sat quietly, without swallowing, and spit out any saliva into a pre-weighed plastic cup for 10 min. Subjects then chewed a piece of wax (Saliva-Check BUFFER, GC, Tokyo, Japan) for 30 s, then spit into another pre-weighed plastic cup. Subjects then continued chewing the wax for an additional 5 min and spit into the cup to collect stimulated saliva. The resting and stimulated saliva were weighed using a balance (Mettler Toledo PL83-S, Columbus, OH, USA) to determine flow rates. Salivary pH was measured with a glass combination electrode (Beckman Coulter A51712; VWR, Atlanta, GA, USA) connected to a pH/ISE meter (Orion 710A, Thermo Scientific, Waltham, MA, USA). The electrode was calibrated with pH 7.00 and pH 4.00 buffer solutions (Fisher Scientific, Waltham, MA, USA). Saliva pH was measured immediately to minimize pH changes. The buffering capacity of stimulated saliva was determined by dispensing one drop onto each of the three test pads (Saliva-Check BUFFER, GC, Tokyo, Japan). After 2 min, the color of each pad was compared to a table provided by the manufacturer to obtain a score from 0 to 4. Only one examiner performed the saliva buffering capacity test throughout the study to reduce inter-examiner differences. Saliva buffering capacity was reported as the sum of scores from the three pads.

### 2.6. Data and Statistical Analysis

Means of health history and questionnaire variables of the ED and control groups were compared using t-tests. Likewise, means of tooth erosion scores, resting and stimulated saliva flow rates, pH, and buffering capacities of the ED and control groups were compared using t-tests. The significance level was 0.05. Counts and proportions of dichotomous variables of the two groups were compared with Fisher’s exact two-tailed tests.

## 3. Results

Table 1 shows demographic characteristics, health history, questionnaire results, DMFT, tooth erosion scores (BEWE), and saliva characteristics of the ED and control groups. Age and weight were not significantly different between the ED and control groups. The ED group had more filled teeth and higher DMFT than the control group, but the numbers of decayed teeth were similar. All control subjects and 11 ED subjects had 28 teeth. The three ED subjects with missing teeth (one, three, and eight teeth, respectively) were missing posterior teeth, except one ED subject missing a lower canine. The tooth erosion scores were not significantly different between the two groups.

Table 2 reports the saliva flow rates, pH, and buffering capacity. The ED group had a significantly lower stimulated saliva flow rate than the control group.

**Figure 2:**
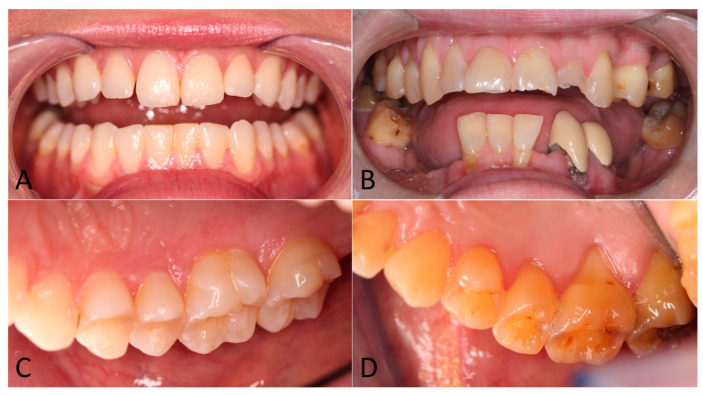
Dentitions from ED subjects without (**A**,**C**) and with tooth erosion (**B**,**D**).

Figure 2 shows intraoral photographs of ED subjects with and without extensive tooth erosion.

**Figure 3:**
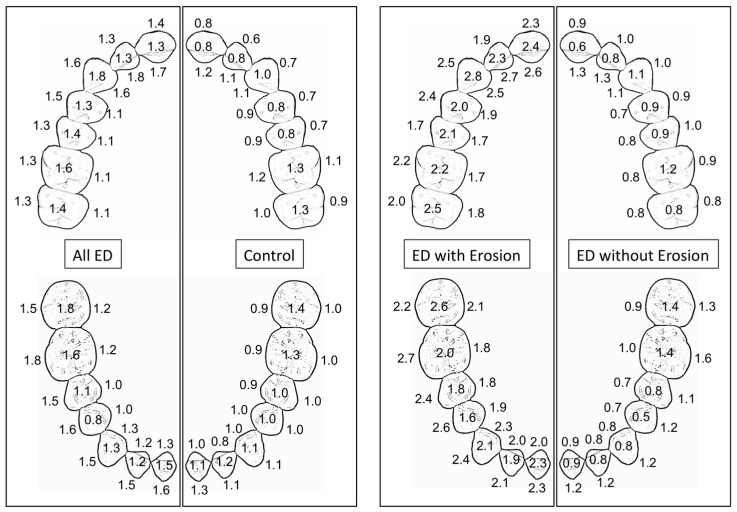
BEWE scores for each surface of the tooth (averaged from the same tooth, **left** and **right** sides of the mouth) from the ED, control, and ED subgroups with and without extensive tooth erosion. No statistics were calculated for ED subgroups.

The differences in tooth erosion patterns among the ED, control, and ED subgroups are visualized in Figure 3, where the average BEWE scores for each tooth surface are summarized. The averaged BEWE tooth erosion scores for the extensive tooth erosion subgroup, the entire ED group, and the control group were 2.16 ± 0.20, 1.38 ± 0.69, and 0.96 ± 0.38, respectively.

## 4. Discussion

Erosive tooth wear becomes a significant oral health problem once it compromises the aesthetics and function of the natural dentition. As seen in Figure 2B, the tooth enamel of the upper incisor teeth was softened and dissolved away, and the edge was chipped off from masticatory force. Figure 2D shows posterior teeth (premolars and molars) with a melted appearance because the softened enamel has worn away and exposed a yellowish layer of dentin beneath. Softening of the enamel surface is an early manifestation of acid erosion. Subsequently, the tooth structures are dissolved layer by layer or by a mechanical insult, resulting in bulk loss of tooth material, which affects the quality of life, and dental treatments to restore form and function are costly. Patients usually experience hypersensitivity when tooth dentin is exposed to the oral environment. Erosive tooth wear from hydrochloric acid in gastric juice has been associated with chronic health issues that patients find difficult to control or disclose. Such extensive tooth erosion can be found when stomach acid is involuntarily regurgitated, such as in GERD, hiatal hernia, or morning sickness; or occurs through chronic vomiting like in bulimia nervosa [24,25].

Systematic reviews and clinical studies have confirmed that tooth erosion is common among patients with eating disorders, with higher odds in those with self-induced vomiting [6,8,19,26,27,28,29,30,31,32]. However, the results of this study rejected our first hypothesis, i.e., no significant difference was found in the averaged BEWE tooth erosion scores between the ED and control groups. After revisiting the data, it was noticed that not every ED subject exhibited tooth erosion. Therefore, the fourteen ED subjects were divided into two subgroups—with extensive tooth erosion (five subjects), and without extensive tooth erosion (nine subjects). ED subjects with extensive tooth erosion had multiple anterior palatal/lingual BEWE scores of 3 (erosion involves dentin) or multiple occlusal and buccal BEWE scores of 3, findings that are characteristic of tooth erosion. ED subjects without extensive tooth erosion had no teeth with a BEWE score of 3 or had only a few teeth with isolated occlusal BEWE scores of 3, a finding that is not characteristic of tooth erosion. It should be noted that the control group also had some tooth surfaces with BEWE scores of 3, mainly on the occlusal surfaces. The biological plausibility alone cannot explain the discrepancy between our findings and what is typically reported by other systematic reviews. We found that six of the nine ED subjects without extensive tooth erosion who reported active purging had an average BEWE of 0.41–1.21, within the same range as the control subjects. Environmental and behavioral factors may explain the extensive tooth erosion found in this study.

The second hypothesis regarding predisposing risk factors of tooth erosion in ED patients was partially accepted. A significantly lower stimulated salivary flow rate was found in the ED group than in the controls, likely a result of antidepressant and antipsychotic medicine (Table 2). Interestingly, the resting salivary flow rate was also lower in the ED group but not significantly so. Only 15 percent of ED subjects reported feelings of dry mouth compared to 20 percent of the controls. In a healthy individual, saliva protects teeth from erosion because it washes away the acid in the mouth and contains mineral components to remineralize damaged tooth structures [14]. Saliva works by diluting and clearing the acid from the mouth, buffering any remaining acid, and supplying the necessary calcium and phosphate minerals for the remineralization of damaged tooth structures [14,33]. Hyposalivation exacerbates the damage of tooth structures exposed to stomach acid [34]. Reduced salivary flow is associated with EDs due to structural changes of salivary glands from prolonged self-induced vomiting and a xerogenic effect from psychotropic medication [10,28,35,36]. A number of commonly prescribed medications with dry mouth as a side effect include anticholinergic agents, antidepressants, antipsychotic agents, diuretic agents, antihypertensive agents, sedative and anxiolytic agents, muscle relaxants, nonsteroidal anti-inflammatory agents, and antihistamines [37]. The acidity (pH) of the resting and stimulated saliva and buffering capacity were not significantly different between the two groups, which agrees with previous studies [15]. Salivary pH can be affected by oral hygiene products and the metabolic activity of oral bacteria from previous meals. Therefore, subjects were asked to refrain from oral hygiene practice and breakfast before their visit. In addition, saliva collection in this study was consistently conducted in the morning to take into account the circadian rhythms. The unstimulated salivary flow rate varies within 24 h and peaks during the late afternoon [38].

Caries experience in ED patients was higher than in healthy individuals [10,28,39]. In this study, the DMFT index and number of filled teeth in the ED group were more than double those of the control group (Table 1). Investigators did not have access to their dental history, so it is unknown whether the filled teeth resulted from erosive tooth wear or dental caries. Impaired salivary function could have played a role in dental caries and tooth erosion.

Another noteworthy observation is that 36 percent of the ED group vs. none of the controls reported having acid reflux (Table 1). Acid reflux symptoms are common in patients with bulimia nervosa and are attributed to frequent irritation of the esophageal mucosa by gastric contents [40]. In addition, repeatedly self-induced vomiting and binge eating vary among ED patients and can lead to spontaneous reflux of gastric contents due to laxity of the lower esophageal sphincter [41,42]. Acid reflux intensifies the process of tooth erosion [43], and might have exacerbated the process in the ED group. As shown in Table 3, 60 percent of ED subjects with extensive tooth erosion reported acid reflux compared to 22 percent of ED subjects without tooth erosion.

We found that individual ED subjects manifested different severities of tooth erosion, similar to previous reports [26,27]. This provides an opportunity to determine potential risk factors or underlying protective mechanisms. Table 3 compares erosion scores, specific health history, saliva analysis, and behavioral components of the ED subgroups with and without extensive tooth erosion [42]. ED subjects with extensive erosion appeared to be older and had a lower body weight and a longer duration of ED than those without extensive erosion. Active purging, psychotropic medication, acidic drink consumption, and saliva characteristics were similar between the two ED subgroups. Dental erosion has been suggested to be more common in ED patients with a longer duration of the disease [8], while other studies reported that the frequency or duration of self-induced vomiting was not linearly associated with tooth erosion [28,44]. Statistical analysis of subgroups was not performed due to the small number of subjects. The findings warrant a larger-scale study in the future.

One of the interesting observations in the current study is the difference in tooth-brushing behavior of the two ED subgroups. Eighty percent of the ED subgroup (four of five subjects) with extensive tooth erosion reported brushing after vomiting. None of the nine subjects in the ED subgroup without extensive tooth erosion brushed after vomiting. This observation suggests that brushing after vomiting may have exacerbated extensive tooth erosion. The literature is inconsistent about this—one study found no difference in the brushing pattern of bulimic patients with or without tooth erosion, whereas others reported an association between brushing immediately after vomiting and severe tooth erosion [26,45,46,47]. Tooth brushing after vomiting is considered a contraindicated behavior [13,28]. The softened tooth surface due to demineralization after an acidic episode is susceptible to the abrading action from tooth brushing; in other words, abrasion of a softened surface exacerbates erosion [34]. Clinical guidelines suggest toothbrushing within an hour of an erosive event as an effective at-home intervention for erosive tooth wear [48]. It should be noted that the only subject in the extensive tooth erosion group who did not report brushing after vomiting had the lowest resting saliva pH (5.7) and the lowest flow rate (0.14 mL/min). An acidic environment from low salivary pH and flow rate might contribute to the extensive tooth erosion manifested in this particular subject.

The results were interpreted with care, realizing that variations among subjects were high. Information about behavioral patterns (such as duration and frequency, active or recovery phase) is sensitive in nature and we collected data based on a questionnaire. The study was conducted at two locations. The ED group was examined at an outpatient rehabilitation center and the controls at a dental school clinical research center. Thus, investigators were not blind to the status of subjects. Control subjects were not matched ethnically with the ED groups, as this variable was not considered in the inclusion/exclusion criteria since no published data have shown an influence of ethnicity on the type of severe tooth erosion associated with oral purging. The sample size in this pilot study was small because of the need for privacy and social stigma surrounding this patient population. However, this pilot study may give new insight into a sensitive and underrepresented population and provide valuable data on which sample size determinations and power calculations can be derived for the planning of future studies to validate these results.

Patients with eating disorders have reported a high level of concern about their dental health and 92% acknowledged having dental problems [13]. Behavioral and medical providers specializing in eating disorder treatment generally are aware of the significance of tooth erosion and oral health care for their patients but may be unaware of associated risk factors [19]. Dentists and dental hygienists are uniquely positioned as the first or only medical professionals who regularly examine patients with EDs [13]. Comprehensive knowledge of intraoral signs and symptoms determined during a dental examination, patterns of tooth erosion, as well as risk factors, can be helpful in early diagnosis of Eds and prevention of the progress of tooth erosion [49].

Preliminary efforts by organized dentistry to determine a guideline for treating and managing dental erosion have recently become available [48]. In addition to modifying tooth brushing behavior as previously mentioned, several acid erosion treatment modalities were shown to be effective in vitro, which can be adapted for clinical practice [48]. These treatment modalities are prescription-level (5000 ppm) or over-the-counter concentrations (1100–1500 ppm) of fluoride in the form of sodium fluoride, casein phosphopeptide–amorphous calcium phosphate paste (CPP–ACP paste) applied for 3 min, 1.23% acidulated phosphate fluoride applied for 4 min, or a combination fluoride and CPP–ACP treatment [50,51,52,53].

This pilot clinical study has a practical focus that will help dental practitioners identify risk factors and recommend proper oral hygiene practices to prevent tooth erosion in patients with EDs. The small sample size is a limiting factor when determining strong associations between these two relevant and emergent themes. Despite lacking statistical significance, we observed some trends that may be useful for practitioners to counsel ED patients on their oral health. Several medications that patients take, especially antidepressants, reduce the salivary flow rate and thus lower the natural defense system for tooth erosion and dental caries. Acid reflux is a comorbidity in ED patients that exacerbates tooth erosion. Lastly, behavior also influences the outcome of tooth erosion. An acidic environment in the mouth, whether from acid reflux, purging, sour food, or acidic medication, will soften tooth structure. Abrasive processes, the most common of which is tooth brushing, will wear away a softened tooth surface. Practitioners should advise ED patients not to brush their teeth after an acid episode. Any mouthwash containing fluoride or calcium should be beneficial to wash away acid and its taste and remineralize tooth structures. Early detection and proper customized home care instructions can prevent the progress of tooth erosion in this population.

Conclusions: The ED group had a significantly lower stimulated saliva flow rate and higher DMFT index than the controls. Although extensive tooth erosion was evident in some ED subjects in this pilot study, the average tooth erosion score of the ED group was not significantly different from the controls. Extensive tooth erosion in EDs may be related to the low stimulated salivary flow. A larger-scale clinical study is necessary to validate these results.

## Figures and Tables

**Table 1:** Demographic information, specific health history, questionnaire results, DMFT, and tooth erosion scores (BEWE) of the eating disorders (ED) and control group.

		ED	Control	*p* Value
Number of subjects (all female)	Sample statistic	14	10	
Age (years)	Mean ± SD	33 ± 13	33 ± 10	0.90 ^(1)^
Weight (lb)	Mean ± SD	177 ± 71	166 ± 21	0.60 ^(1)^
Ethnicity	Caucasian	10	2	
	Black	2	8	
	Others	2		
Duration of ED (years)	Mean ± SD	17 ± 13	0	
Active purging (vomiting)	percent	64	0	0.002 ^(3)^
Taking antidepressant	percent	64	10	0.02 ^(3)^
Taking antipsychotic	percent	21	0	0.24 ^(3)^
Have acid reflux	percent	36	0	0.06 ^(3)^
Take acidic drinks	percent	93	100	0.99 ^(3)^
Brushing after purging (vomiting)	percent	31	n/a	n/a
Feeling of dry mouth	percent	15	20	0.99 ^(3)^
DMFT	Mean ± SD	11.57 ± 5.83	4.70 ± 4.72	0.006 ^(1)^
Number of decayed teeth	Mean ± SD	3.64 ± 3.89	2.30 ± 2.91	0.37 ^(1)^
Number of filled teeth	Mean ± SD	7.07 ± 4.18	2.40 ± 4.55	0.02 ^(1)^
Number of missing teeth	Mean ± SD	1.09 ± 2.47	0	n/a
Tooth erosion score (BEWE)	Mean ± SD	1.38 ± 0.69	0.96 ± 0.38	0.08 ^(2)^

Notes: ^(1)^ Means of health history and questionnaire variables of the ED and control groups were compared using *t*-tests. The significance level was 0.05. ^(2)^ Means of tooth erosion scores of the ED and control groups were compared using *t*-tests. The significance level was 0.05. ^(3)^ Counts and proportions of dichotomous variables of the two groups were compared with Fisher’s exact two-tailed tests. The significance level was 0.05.

**Table 2:** Saliva flow rates, pH, and buffering capacity of the eating disorders (ED) and control group (Mean ± SD).

	ED	Control	*p* Value
Number of subjects	12	10	
Resting saliva flow rate (mL/min)	0.44 ± 0.26	0.61 ± 0.35	0.22
Stimulated saliva flow rate (mL/min)	1.80 ± 0.75	3.01 ± 1.30	0.02
Resting saliva pH	6.77 ± 0.44	6.92 ± 0.42	0.41
Stimulated saliva pH	7.25 ± 0.29	7.30 ± 0.30	0.66
Buffering capacity (stimulated saliva)	9.67 ± 2.81	10.80 ± 1.81	0.29

Notes: Resting and stimulated saliva flow rates, pH, and buffering capacities of the ED and control groups were compared using *t*-tests. The significance level was 0.05.

**Table 3:** Characteristics of ED subgroups with or without extensive tooth erosion (Mean ± SD, no statistics calculation).

	ED with Extensive Tooth Erosion	ED without Extensive Tooth Erosion
Tooth erosion score (BEWE)	2.16 ± 0.20	0.95 ± 0.41
Number of teeth with BEWE score ‘3’ per subject	14.80 ± 4.02	0.22 ± 0.67
Age (years)	41 ± 6	32 ± 14
Weight (lb)	146 ± 42	202 ± 77
Duration of ED (years)	23 ± 4	14 ± 15
Active purging (vomiting) (%)	60	67
Taking antidepressants (%)	80	56
Taking antipsychotic (%)	20	22
Have acid reflux (%)	60	22
Take acidic drinks (%)	100	89
Brushing after purging (vomiting) (%)	80	0
Feeling of dry mouth (%)	20	13
Resting saliva flow rate (mL/min)	0.26 ± 0.10	0.53 ± 0.27
Stimulated saliva flow rate (mL/min)	1.70 ± 0.54	1.86 ± 0.87
Resting saliva pH	6.46 ± 0.60	6.92 ± 0.26
Stimulated saliva pH	7.20 ± 0.23	7.27 ± 0.33
Buffering capacity	11.75 ± 0.50	8.63 ± 2.92

## Data Availability

Data is contained within the article.
